# Membrane insertion and secretion of the Engrailed-2 (EN2) transcription factor by prostate cancer cells may induce antiviral activity in the stroma

**DOI:** 10.1038/s41598-019-41678-0

**Published:** 2019-03-26

**Authors:** Natasha Punia, Monika Primon, Guy R. Simpson, Hardev S. Pandha, Richard Morgan

**Affiliations:** 1 Faculty of Health and Medical Sciences, University of Surrey, Guildford, UK; 2 Institute of Cancer Therapeutics, Faculty of Life Sciences, University of Bradford, Bradford, UK

## Abstract

Engrailed-2 (EN2) is a homeodomain-containing transcription factor that has roles in boundary formation and neural guidance in early development, but which is also expressed in a range of cancers. In addition to transcriptional regulation, it is secreted by cells and taken up by others through a mechanism that is yet to be fully elucidated. In this study, the distribution of EN2 protein in cells was evaluated using immunofluorescence with a set of antibodies raised against overlapping epitopes across the protein, and through the use of an EN2-GFP construct. MX2 expression in primary prostate tumors was evaluated using immunohistochemistry. We showed that EN2 protein is present in the cell membrane and within microvesicles that can be secreted from the cell and taken up by others. When taken up by normal cells from the stroma EN2 induces the expression of MX2 (MxB), a protein that has a key role in the innate immune response to viruses. Our findings indicate that EN2 secretion by tumors may be a means of preventing viral-mediated immune invasion of tissue immediately adjacent to the tumor.

## Introduction

Engrailed is a homeodomain-containing transcription factor originally identified through the effects of an inactivating mutation in *Drosophila* that resulted in a failure of the border between the anterior and posterior wing compartments^1^. The human genome encodes 2 homologues of this gene, Engrailed-1 and Engrailed-2 (EN2), which have similar functions in early development^2^, although of the two EN2 has been more extensively characterized^3^. Both EN1 and EN2 have a role in determining the midbrain/hindbrain border in vertebrates^4,5^, and their expression is required subsequently for development and continued survival of the mesencephalic dopaminergic neurons^6–8^.

Engrailed proteins primarily function as transcriptional repressors through an interaction with the co-repressor protein groucho^9^, although they have also been shown to regulate translation through binding to the eukaryotic translation initiation factor 4E^10^. In addition, there is evidence that EN2 can be secreted from cells in a process that is dependent on a peptide sequence within the DNA-binding homeodomain region^11–14^, and that in the developing brain this gives rise to an extracellular gradient of EN2 protein that can be taken up by temporal retina and nasal axons resulting in attraction and repulsion, respectively^15^. These effects on axonal migration appear to be mediated at the level of translational regulation exerted by the internalized EN2 protein^15^.

In addition to a role in neural development, early studies also indicated that EN2 had an oncogenic function in breast cancer cells as its forced expression in non-tumorigenic mammary cell lines resulted in a number of malignant characteristics including a loss of cell-to-cell contact and a failure to differentiate in response to lactogenic hormones^16^. More recently it has been shown that EN2 is expressed in a number of other tumor types, including those of the prostate^17–19^ and bladder^20^. The presence of EN2 protein in the urine of prostate and bladder cancer patients has been shown to have both diagnostic and prognostic value as the concentration of urinary EN2 correlates with both tumor size and grade^17–21^.

Despite the highly unusual properties of EN2 and its importance both in development and in cancer, there is in fact still relatively little known of the underlying molecular mechanisms. For example, it is unclear whether EN2 is actually present in the membrane of cells, or whether it is secreted or taken up by an active mechanism. There is also no direct evidence that it can be taken up by neighboring cells, or that in doing so it alters the behavior of these cells. In this study, we show that EN2 is indeed present in the membrane of cancer cells and that it is secreted through an active mechanism that is dependent on vesicle formation, and that cells that take up exogenous EN2 protein undergo distinct changes in behavior that could profoundly influence tumor development through modification of the tumor microenvironment.

## Methods

### Cell culture

Human prostate adenocarcinoma PC-3 cells, human prostate carcinoma LnCaP and DU145 cells, and human normal prostate stroma/fibroblast WPMY-1 cells were obtained from the American Type Culture Collection (ATCC) and cultured as previously described^22^. The human melanoma cell line SKMEL5 (HTB-70) was obtained from the ATCC via LGC Standards Ltd and cultured according to ATCC protocols. The promyelocytic leukemia derived cell line HL60 was also obtained from the ATCC (via LGC Standards Ltd) and was cultured as previously described^23^. Growth conditions for all the cell lines used are presented in Supplementary Table 1.

### Production of transient and stable GFP-EN2 clones

Plasmid transfections for forced expression of EN2 tagged with GFP were carried out following the manufacturer’s instructions using Lipofectamine® 2000 (Life Technologies, UK) for DU145 cells (6 μl of reagent per 1 μg of DNA) or ViaFect™ (Promega, UK) for the other cell lines, using the following reagent(μl):DNA(μg) ratios: PC3 (3:1), LnCaP (5:1), WPMY-1 (6:1), SKMEL-5 (5:1). During transfection, cells were starved by reducing fetal bovine serum levels in fresh media from 10% to 0.5%. Cell lines were transfected with DNA plasmid created using Myc-DDK tagged ORF clone of Homo sapiens EN2 as transfection ready DNA (accession number: NM_001427) and PrecisionShuttle mammalian vector with N-terminal mGFP (OriGene Technologies, USA), which enabled the expression of N-terminal GFP-tagged EN2. As a control, cell lines expressing GFP only were created in the same way using the PrecisionShuttle mammalian vector with N-terminal mGFP (OriGene Technologies, USA) alone.

### EN2 silencing in PC3 GFP-EN2 stable cells

siRNA transfections were carried out using siPORTTM NeoFXTM transfection reagent (Invitrogen, UK) following the manufacturer’s protocol. Briefly, EN2 siRNA (4676; mRNA sequence accession number NM_001427.3, targeted exon 2, siRNA location 1110 bp) or negative control siRNA diluted in Optimem and siPORTTM NeoFXTM transfection reagent diluted in Optimem were mixed at 1:1 ratio, added to PC3 cells and EN2 protein detected 48 h after transfection using immunocytochemistry.

### Quantitative real-time PCR (RT-qPCR)

Total RNA was isolated using the RNeasy Plus Micro Kit (Qiagen, UK) followed by a two-step cDNA synthesis reaction using nanoScript Reverse Transcription kit (Primer design Ltd, UK) with annealing step preceding the extension step, all according to the manufacturer’s protocol. The mRNA expression of *EN2*, *GAPDH* and *MX2* was quantified using the Stratagene Mx3005P qPCR system (Agilent Technologies, USA) with SYBR Green fluorescence technology with the following primer sequences: EN2 NM_001427 F: GAACCCGAACAAAGAGGACA and R: CGCTTGTTCTGGAACCAAAT, GAPDH NM_002046.5, F: GAACCCGAACAAAGAGGACA and R: CGCTTGTTCTGGAACCAAAT, and MX2 NM_002463: F: AAGCAGTATCGAGGCAAGGA and R: TCGTGCTCTGAACAGTTTGG. *EN2* and *MX2* expressions were normalised to *GAPDH* using the ΔCt relative quantification method.

### EN2 protein detection in cell lines

Immunocytochemistry was used to demonstrate the  presence of EN2 in cells. Cell lines were grown overnight in an 8-chambered polystyrene culture treated glass slides (BD Biosciences, UK), fixed with 4% paraformaldehyde (Sigma, UK) and washed with PBS three times. Next, the cells were incubated with 10% horse serum (Jackson ImmunoResearch, USA) for 20 minutes and further with anti-EN2 goat polyclonal IgG primary antibody (Abcam, UK) diluted 1:100 in PBS/1% BSA at room temperature for 2 hours or overnight at 4 °C. A ‘no primary antibody’ sample was included containing PBS/1% BSA alone for use as a negative control. After primary antibody incubation, the cells were washed three times with PBS and incubated with donkey anti-goat secondary antibody (Abcam, UK) at 1:200 in the dark for 45 minutes. After incubation, cells were washed three times with PBS, chambers removed and slides mounted using propidium iodide (PI) Vectashield® mounting medium (Vector Laboratories, USA). The slides were imaged at X40 magnification using the Nikon A1M confocal microscope and NIS elements acquisition software (Nikon, UK). Images were processed with ImageJ.

### EN2 staining using epitope-specific antibodies

A panel of 19 polyclonal sheep antibodies, Ab2 - Ab33 (kindly provided by Bioventix, UK, as part of a collaborative project) (Supplementary Table 2), were made against peptides covering the whole length of the EN2 protein. Each peptide was 20 amino acids long and overlapped its neighboring peptides by 10 amino acids. These antibodies were generated in sheep using a nested peptide series conjugated to the metalloprotein, KLH. Immunocytochemical staining was performed as described above, except 5% horse serum was used for blocking and donkey anti-sheep secondary antibody (Abcam, UK) at 1:10,000 dilution was used for visualisation.

### Time-lapse confocal microscopy

PC3 cells were seeded in a 4-chamber glass-bottom dish (MatTek, USA) and 24 hours later transfected with 0.5 μg of either GFP-EN2 or GFP (control) plasmids as described above. After further 24 h incubation, the media was changed to Gibco® FluoroBrite™ DMEM (Life Technologies, UK) in order to enhance the fluorescent signal. GFP fluorescence was detected using confocal microscopy. Time-lapse mode was selected and the areas to be imaged were chosen by focusing on the cells as well as labelling and setting the X/Y parameters. Images were set to be taken every 5 minutes over 24 hours. Fold difference in fluorescence from T0 was calculated and plotted over time.

### EN2 direct co-culture assay

To investigate the intercellular transfer of EN2, stable PC3 LifeAct® (Ibidi, Germany) expressing clones were created as described above. F-actin was visualized in the cells to reveal the cytoskeleton without compromising cellular processes. These cells were then directly co-cultured with PC3 EN2GFP (green fluorescence) or PC3 GFP cells in 6-well plates at half the normal density in DMEM culture media. Images were taken after 72 h using a fluorescence microscope at X20 or X40 magnification. The assay was repeated and images taken after 96 h with addition of NucBlue® Live ReadyProbes® Reagent to demarcate the nucleus.

### Immunohistochemistry

MX2 expression was investigated in prostate cancer and normal prostate using a 3 µm-thick, formalin fixed, paraffin embedded tissue array (PR2085a, US Biomax, Rockville, MD, USA), with a rabbit polyclonal MX2 antibody (Abcam 224479) diluted 1:100 and the ABC detection method with peroxidase block (DakoCytomation). Antigen retrieval was performed using pH 6.0 citrate/EDTA buffer (DakoCytomation) and heating in a microwave for 23 minutes. DAB Peroxidase (HRP) Substrate Kit (Vector Laboratories) was used in the final detection step and images taken under a light microscope at × 40 magnification.

### Statistical analysis

Statistical analyses were performed using GraphPad-PRISM software (CA, USA) based on a minimum of two independent experiments using Student’s t-test for comparison between two groups. Significance was scored as: ****P < 0.0001; ***P < 0.001; **P < 0.01; *P < 0.05.

## Results

**Figure 1:**
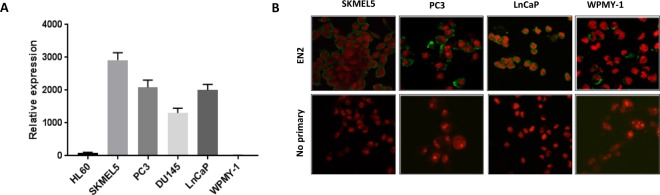
Engrailed-2 (EN2) expression in various cancer and normal derived cell lines. (**A**) qRT-PCR for *EN2* mRNA expression, normalised to expression of the *GAPDH* house-keeping gene (x1000000). (**B**) Fluorescent micrograph of cancer cells stained with an anti-EN2 antibody. EN2 staining is shown in green (FITC-labeled secondary antibody). Cell nuclei are stained red (propidium iodide), images were taken by confocal microscopy at x 40 magnification.

**Figure 2:**
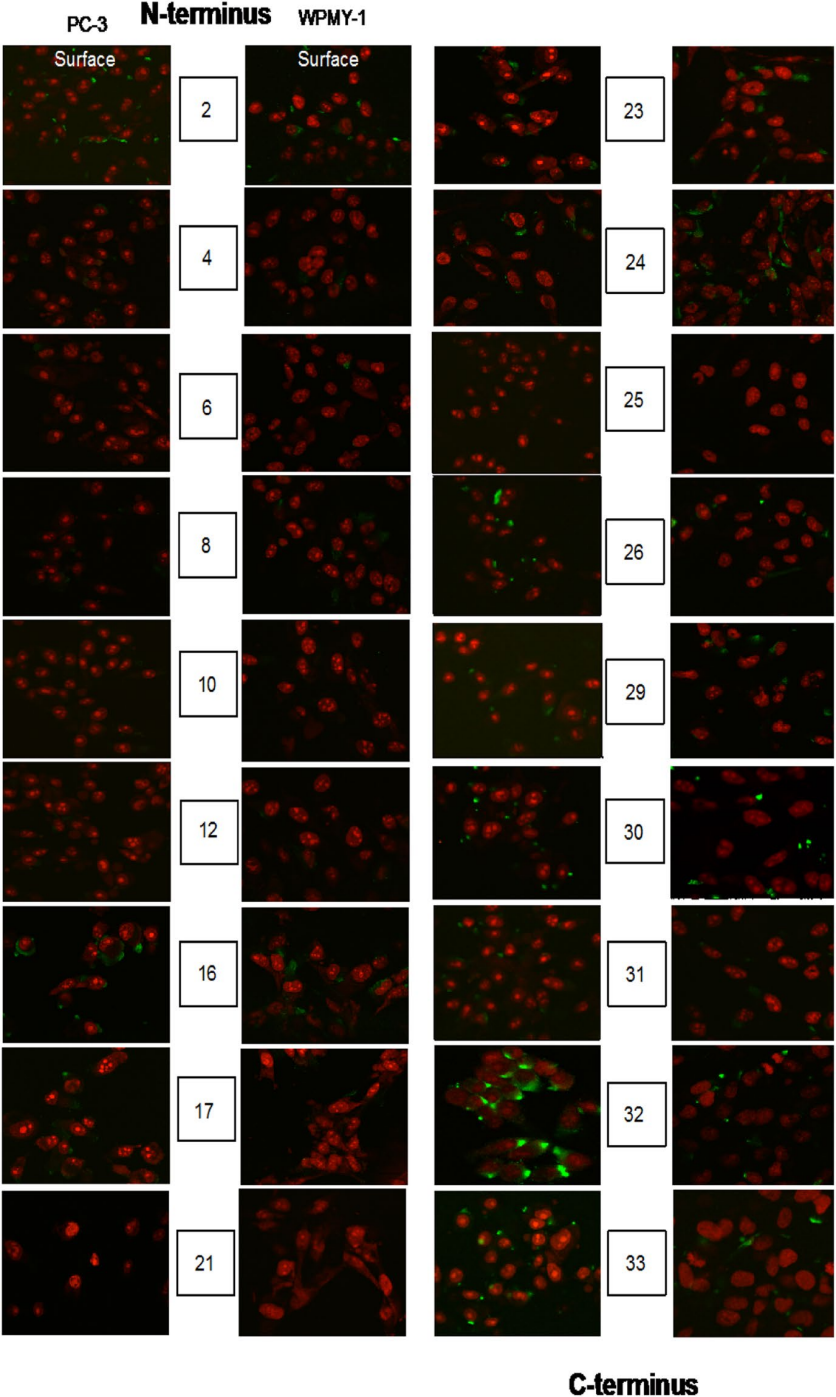
EN2 peptide-specific antibody panel for antibody-drug conjugate. A panel of sheep antibodies that target 20 amino acid (aa) immunogens across the length of the EN2 protein were used for cell surface fluorescence staining (green) with propidium iodide nuclear staining (red), 10X confocal microscopy.

In order to understand more about the role of EN2 in cancer, we began by studying its expression in cells lines derived from tumors and normal tissue. RT-QPCR revealed that promyelocytic leukemia derived HL60, melanoma derived SKMEL5, and the prostate cancer-derived cell lines DU145, PC3, and LNCaP, all express high levels of *EN2*, whilst WPMY-1 (a cell line derived from normal prostate fibroblasts) showed only very low levels of expression (Fig. 1A). Fluorescent confocal microscopy with an anti-EN2 antibody revealed that EN2 protein was present in the membrane of SKMEL5, PC3 and LnCaP cells (Fig. 1B). Given the apparent membrane expression of EN2, we sought to determine which parts of the EN2 protein were present on the outside of the cell, as this could help to identify potential target antigens for antibody therapy. In order to do this, a series of antibodies were raised in sheep using 20 amino acid peptides that overlapped each other by 10 amino acids, and which together covered the whole length of the EN2 protein. When the antibodies were used to stain PC3 cells that had not been treated to make the membrane permeable, only antibody 32 (Ab32), corresponding to the C-terminal most portion of EN2, gave a strong signal compared to WPMY-1 cells that were used as a negative control (Fig. 2).

**Figure 3:**
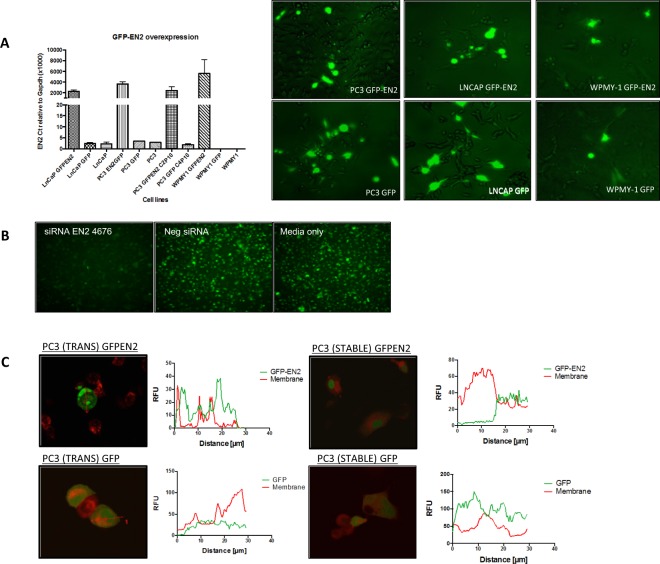
EN2 localisation confirmed using GFP-EN2 de novo expression. (**A**) qRT-PCR of *GFP-EN2* overexpression with 1μg plasmid DNA (left), these were analysed by fluorescence microscopy 48 h after transfection (right). (**B**) GFP-EN2 stably expressing cells treated with EN2 siRNA, negative scrambled siRNA or media only imaged at x20 magnification using a fluorescence microscope and probed with goat anti-EN2 antibody, 48hrs after transfection. (**C**) PC3 cells transfected transiently and stably with GFP-EN2 plasmid DNA were then co-stained with wheat germ agglutinin membrane stain (red), these were imaged and analysed using the confocal microscopy software to show co-localisation (40X magnification), the arrows on the images depict the cell through which the measurement was taken.

To further explore the cellular distribution of EN2 we made an EN2-green fluorescent protein (GFP) construct, which produced *GFP-EN2* mRNA and GFP-EN2 protein when transfected into cells (Fig. 3A). The identity of this protein was confirmed in the transfected cells through specific knockdown using an anti-EN2 siRNA (Fig. 3B). As with the endogenous EN2 protein, a significant proportion of GFP-EN2 was found to be present in the cell membrane upon confocal microscopy. GFP alone was only located in the cytoplasm (Fig. 3C).

**Figure 4:**
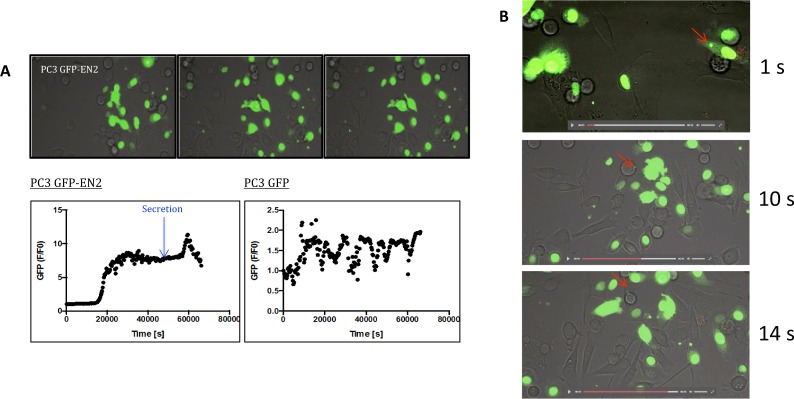
Microvesicle secretion of EN2 was only observed in PC-3 prostate cancer cells. (**A**) Stills of time-lapse video of transiently overexpressed GFP-EN2 in PC3 cancer cells (top) and the fold difference in fluorescence from T0 plotted over time (bottom). (**B**) Stills from a time-lapse video of PC3 cells transiently overexpressing EN2-GFP (green fluorescence), showing the formation of intracellular vesicles (1 second, red arrow), its uptake by a non-adherent cell (10 seconds, red arrow), and the subsequent adhesion and migration of that cell (14 seconds, red arrow).

**Figure 5:**
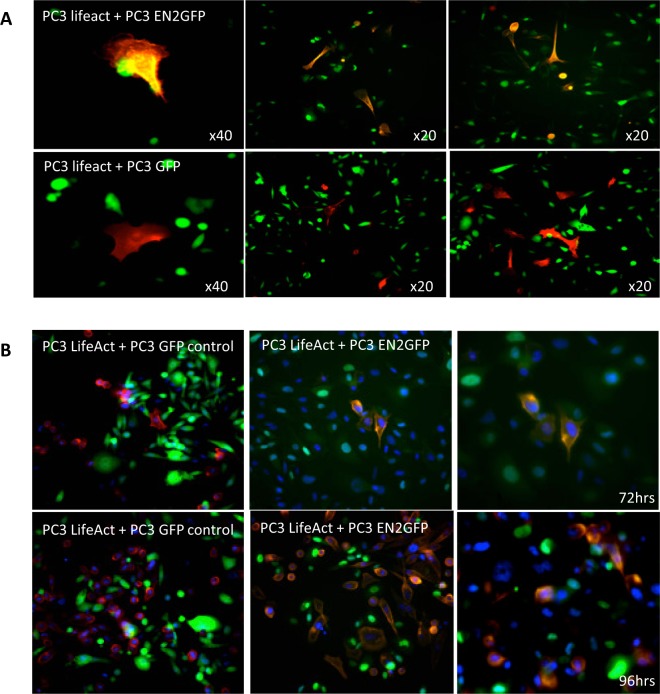
EN2 co-culture assay. (**A**) PC3 EN2-GFP and GFP control stable cell lines (green) were co-cultured with either PC-3 or WPMY-1 stable LifeAct (red) cell lines for 72 h and images were taken at x20 or x40 magnification. (**B**) The assay was repeated after 96 h with additional NucBlue nuclear stain (blue). EN2 has a granular distribution within the cytoplasm.

Following the transfected cells over time revealed that GFP-EN2 transfected PC3 cells began to secrete GFP-EN2 protein in discrete bodies (Video). This behavior was not observed for LNCaP, WPMY-1, or SKMEL-5 cells transfected with GFP-EN2, or in any of these cell types transfected with GFP alone (data not shown). The fluorescence was quantified over this time period, revealing that GFP-EN2 secretion from PC3 cells began shortly before a large spike in fluorescence (Fig. 4A, blue arrow). The imaging of single cells at higher magnification confirmed the production of GFP-EN2 containing bodies and the uptake of these bodies by other cells (Fig. 4B). We further assessed the mechanism of uptake of EN2 by co-culturing PC3 or WPMY-1 cells transfected with EN2-GFP with untransfected cells labelled using LifeAct (red). This indicated that EN2-GFP can indeed move between cells, although this process appears to require cell to cell contact (Fig. 5A). Once internalized, the imported EN2-GFP protein is restricted to specific areas of the cell within the cytoplasm (Fig. 5B).

**Figure 6:**
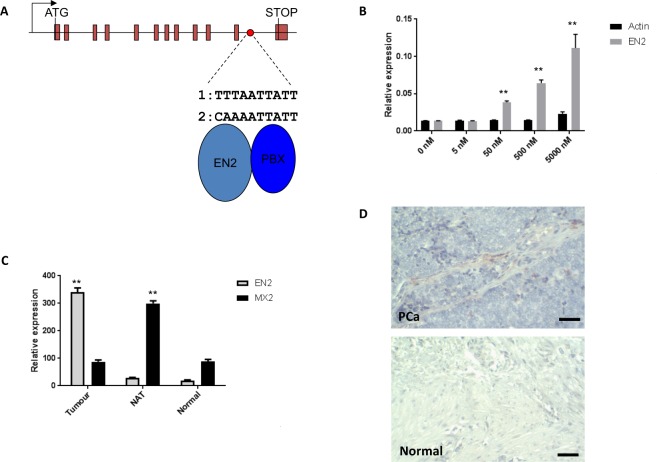
Exogenous EN2 protein induces the expression of *MX2* in WPMY-1 cells. (**A**) Location of a putative EN2/PBX binding site in the genomic sequence of *MX2*. (**B**) *MX2* expression in WMPY-1 cells treated with recombinant EN2 or actin protein. (**C**) Expression of *MX2* and *EN2* in normal prostate tissue (‘Normal’), prostate tumours and normal tissue adjacent to tumours (‘NAT’). Expression is shown relative to the house keeping gene *GAPDH* (x1000). Error bars represent the SEM for three independent experiments, *p < 0.05, **p < 0.01. (**D**) MX2 protein expression in prostate tumours. Immunohistochemical staining of a prostate tumour section (PCa) and normal prostate tissue (Normal). MX2 staining (brown) is present in the stroma surrounding tumour cells but not in normal prostate stroma. Scale bar: 80 μm.

The secretion and uptake of EN2 protein suggests the possibility that normal prostate cells adjacent to prostate tumour cells could be exposed to sufficiently high levels of EN2 to change their transcriptional states. To address this possibility, we exposed WMPY-1 cells, which are derived from non-malignant prostate fibroblast cells, to purified EN2 protein and used microarray to look for genes that show a stepwise increase or decrease in expression with increasing amounts of EN2 protein. This approach revealed only a single gene that showed such a dose-response relationship, MX dynamin-like GTPase2 (*MX2*, also known as *MxB*), which encodes a dynamin related protein^24^. The *MX2* genomic region contains an EN2/PBX binding site within a intronic region (Fig. 6A), and qPCR analysis of WMPY-1 cells treated with EN2 protein revealed a 12-fold increase in transcription stepwise over 4 logs of increasing protein concentration (Fig. 6B). Analysis of *MX2* expression in primary prostate tumours, normal prostate tissue and normal tissue adjacent to tumours (NAT) revealed high levels of *MX2* expression only in NAT (Fig. 6C). Correspondingly, immunohistochemical analysis of prostate tumours and normal prostate tissue revealed that MX2 protein is only present in the stroma surrounding prostate tumours and not in normal prostate tissue (Fig. 6D).

## Discussion

It is now generally accepted that a key part of tumor biology is the reactivation and re-tasking of developmental pathways and genes to support a malignant phenotype. EN2, a transcription factor that can be secreted from cells and taken up by others^3^, provides a particularly striking example of this phenomenon. In this study we have sort to elucidate the mechanisms that underlie EN2 protein secretion and uptake. Our findings indicate that EN2 protein can be secreted from cancer cells via microvesicles and that it can be taken up by other, non-EN2 expressing cells. This was restricted to PC3 cells, and was not observed in the other prostate cancer-derived cell lines tested, a finding that possibly reflects the very wide variation in the concentration of secretory vesicles found in the plasma of prostate cancer patients^25^. In addition to secretory vesicles, EN2 protein is present in the plasma membrane of cells, and although further work is needed to understand the exact mechanism of insertion and its relationship with the phospholipid bilayer, our findings indicate that part of the EN2 protein is exposed on the outside of the cell, and hence could potentially act as a target in antibody-directed therapy.

In addition to being taken up by other cancer cells, we found that EN2 can be taken up by cells derived from fibroblasts that would normally form part of the stroma surrounding tumors. This concurs with the findings of earlier studies^11–15^, but we have extended this to assess how it might influence the behavior of normal cells. We show that externally supplied EN2 protein can cause a specific, dose-dependent increase in *MX2* gene expression in fibroblast cells. Furthermore, while neither MX2 protein nor RNA was detected in prostate tumors, or indeed in normal prostate cells in the absence of cancer, both are detected at high levels in ‘normal’ stromal cells adjacent to tumors. These findings indicate that EN2 secretion by prostate tumors can cause MX2 to be expressed by the surrounding stroma. MX2 is a dynamin-related GTPase that is primarily located at the cytoplasmic interface of nuclear pores and has a role in nuclear transport and cell cycle regulation, as expression of a dominant negative (GTPase inactive) version blocks nuclear import and prevents G1/M transition^26^. Its primary cellular function seems to be the inhibition of the viral life cycle as it can block the assembly of multiple viruses^27^ including the vesicular stomatitis virus^28^, and prevent the nuclear entry of HIV^29^. The relevance of EN2-induced MX2 expression in cells adjacent to the tumor is unclear, although it could be advantageous for the tumor to prevent viral infection of stromal cells that would otherwise generate a localized immune response. To date there are no reported studies that directly support this hypothesis, although a single nucleotide polymorphism (SNP) in the *MX2* gene has been found to be associated with a reduced overall risk of melanoma^30^.

## Conclusion

**Figure 7:**
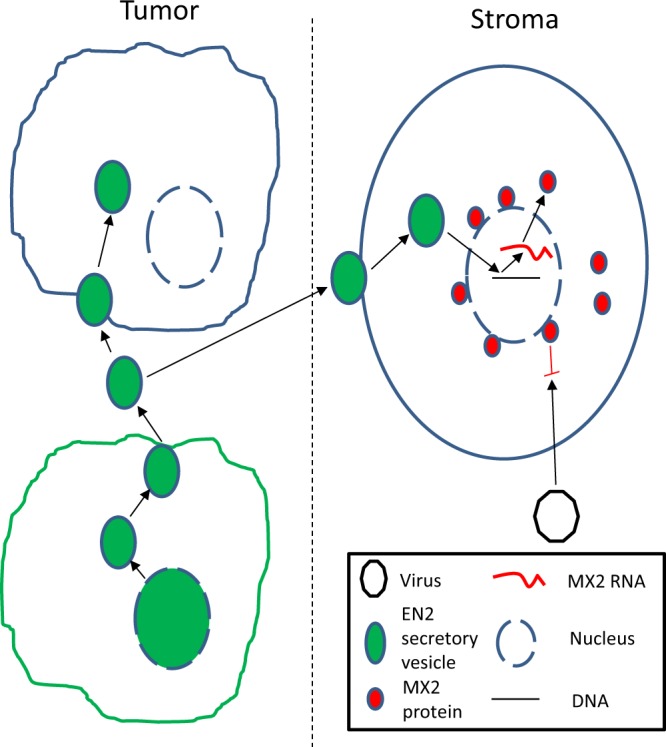
EN2 secretion may mediate anti-viral changes in the stroma. In EN2 (green) expressing cancer cells EN2 protein is present in both the nucleus and the membrane, and within microvesicles. These microvesicles can be secreted and taken up by other cancer cells and non-cancer cells in the stroma. In the latter, EN2 activates the transcription of MX2 (red), a key component of the innate defense against viral infection.

Taken together, our findings suggest that EN2 secretion and uptake represent a novel mechanism by which tumors can influence the behavior of surrounding cells. EN2 can be secreted from cells via microvesicles that originate in the nucleus and also be inserted into the membrane, possibly through the same mechanism. These vesicles can be taken up by other cancer cells, and by stromal cells (although a different mechanism might also be involved). In the latter, EN2 drives expression of the *MX2* gene, a key component of the innate defense against viral infection (Fig. 7), which in turn helps prevent a viral-mediated localized immune response that could indirectly expose the tumor to immune-mediated killing. Further study of the action of EN2 and the significance of MX2 expression in the stroma should help clarify the significance of this protective mechanism.

## Supplementary information

Supplementary file

Video

## Data Availability

All data generated or analysed during this study are included in this published article [and its Supplementary Information file].
