# Supplementary material for: Membrane insertion and secretion of the Engrailed-2 (EN2) transcription factor by prostate cancer cells may induce antiviral activity in the stroma
Source: Sci Rep. 2019 Mar 26;9:5138. doi: 10.1038/s41598-019-41678-0 (PMC6435720; doi:10.1038/s41598-019-41678-0)
Supplement: Supplementary file 1 — Supplementary file [file 41598_2019_41678_MOESM1_ESM.pdf]

Membrane insertion and secretion of the Engrailed-2 (EN2) transcription factor by prostate cancer cells may induce antiviral activity in the stroma

Running title: Engrailed-2 secretion in prostate cancer

Natasha Punia[1], Monika Primon[2], Guy R Simpson[1], Hardev S Pandha[1], Richard Morgan[2*]

1. Faculty of Health and Medical Sciences, University of Surrey, Guildford, UK

2. Institute of Cancer Therapeutics, Faculty of Life Sciences, University of Bradford, UK

Supplementary Table 1

**Cell line**	**Media and supplements**	**Environmental conditions**
HL-60	IMDM + 20% FBS + 1% P/S	5% CO_2, 37˚C
SK-MEL-5	MEM + 10% FBS + 1% glutamate +1% P/S + 1% sodium pyruvate	5% CO_2, 37˚C
PC3	F-12K +10% FBS + 1 %P/S	5% CO_2, 37˚C
Du145	DMEM + 10% FBS + 1% glutamate + 1% P/S	5% CO_2, 37˚C
LnCaP	RPMI-1640 + 10% FBS + 1% glutamate + 1% P/S	5% CO_2, 37˚C
WPMY-1	DMEM + 10% FBS + 1% glutamate + 1% P/S	10% CO_2, 37˚C

Supplementary Table 1: The media, supplements and environmental conditions used for each cell line.

Membrane insertion and secretion of the Engrailed-2 (EN2) transcription factor by prostate cancer cells may induce antiviral activity in the stroma

Running title: Engrailed-2 secretion in prostate cancer

Natasha Punia[1], Monika Primon[2], Guy R Simpson[1], Hardev S Pandha[1], Richard Morgan[2*]

1. Faculty of Health and Medical Sciences, University of Surrey, Guildford, UK

2. Institute of Cancer Therapeutics, Faculty of Life Sciences, University of Bradford, UK

Supplementary Table 2

Antibody name	Epitope location (amino acid)	Peptide sequence (N'-C' terminal)
Ab33	330 - 310	AVHLMAQGLYNHSTTAKEGK
Ab32	320 - 300	KKATGNKNTLAVHLMAQGLY
Ab31	310 - 290	IWFQNKRAKIKKATGNKNTL
Ab30	300 - 280	ELSLNESQIKIWFQNKRAKI
Ab29	290 - 270	EQRRQSLAQELSLNESQIKI
Ab26	260 - 240	NKEDKRPRTAFTAEQLQRLK
Ab25	250 - 230	RSRKPKKKNPNKEDKRPRTA
Ab24	240 - 220	RYSDRPSSGPRSRKPKKKNP
Ab23	230 - 210	MLWPAWVYCTRYSDRPSSGP
Ab21	210 - 190	DSDSSQAGANLGAQPMLWPA
Ab17	170 - 150	PGDGEGGSKTLSLHGGAKKG
Ab16	160 - 140	GPLPAAGSDSPGDGEGGSKT
Ab12	120 - 100	GGEGGASGAEGGGGAGGSEQ
Ab10	100 - 80	RRKDAGTCCAGAGGGRGGGA
Ab8	80 - 60	QHPHRITNFFIDNILRPEFG
Ab6	60 - 40	DTGRRRALMLPAVLQAPGNH
Ab4	40 - 20	PESSPGGGSGGGGGSSPGEA
Ab2	20 - 0	MEENDPKPGEAAAAVEGQRQ

Supplementary Table 2: Peptides used to raise sheep polyclonal IgG antibodies against the EN2 protein.

Membrane insertion and secretion of the Engrailed-2 (EN2) transcription factor by prostate cancer cells may induce antiviral activity in the stroma

Running title: Engrailed-2 secretion in prostate cancer

Natasha Punia[1], Monika Primon[2], Guy R Simpson[1], Hardev S Pandha[1], Richard Morgan[2*]

1. Faculty of Health and Medical Sciences, University of Surrey, Guildford, UK

2. Institute of Cancer Therapeutics, Faculty of Life Sciences, University of Bradford, UK

Legend for video

EN2-GFP secretion and reuptake from PC3 cells transiently overexpressing a GFP-EN2 expression construct
